# The Medical Profession in Rome

**Published:** 1879-08

**Authors:** 


### THE MEDICAL PROFESSION IN ROME.

Rome now contains six hundred and eighty-two persons exercising the various branches of our art: one hundred and eighty-three physicians and surgeons, one hundred and forty-two physicians, sixty-six surgeons, seventy-four apothecaries, twenty-three veterinary surgeons, one hundred and forty-five midwives, twenty dentists, and twenty-nine phlebotomists. The presence of so many professional bleeders is pretty conclusive proof that the lancet is not, as with us, repudiated by Italian physicians. That venesection is largely practised is shown by the fact that there are more phlebotomists than dentists.

### THE ADMINISTRATION OF GELSEMIUM.

According to Professor Massini, the cases in which gelsemium produces most benefit are those of simple rheumatic neuralgia of the alveolar branches of the trigeminus; in those it rarely fails. It also sometimes relieves the pain remaining after the stopping of a carious tooth. Where there is any inflammatory affection of the bone or periosteum no good can be expected from the remedy. The medicine may, if necessary, be repeated several days in succession, the active principle rapidly passing off by the kidneys.

